# Neurodegenerative phosphoprotein signaling landscape in models of SCA3

**DOI:** 10.1186/s13041-020-00723-0

**Published:** 2021-03-19

**Authors:** Anna S. Sowa, Taissia G. Popova, Tina Harmuth, Jonasz J. Weber, Priscila Pereira Sena, Jana Schmidt, Jeannette Hübener-Schmid, Thorsten Schmidt

**Affiliations:** 1 Institute of Medical Genetics and Applied Genomics, University of Tuebingen, Calwerstrasse 7, 72076 Tuebingen, Germany; 2 Centre for Rare Diseases, University of Tuebingen, 72076 Tuebingen, Germany; 3 Center for Applied Proteomics and Molecular Medicine, College of Science, George Mason University, Manassas, VA USA; 4 Department of Human Genetics, Ruhr-University Bochum, Universitaetsstrasse 150, 44801 Bochum, Germany

**Keywords:** Spinocerebellar ataxia type 3 (SCA3), Machado-Joseph disease (MJD), Ataxin-3 (ATXN3), RPMA, Neurodegeneration, pERK, AKT (PKB), mTOR, Phosphoprotein

## Abstract

Spinocerebellar ataxia type 3 (SCA3) is a rare neurodegenerative disorder resulting from an aberrant expansion of a polyglutamine stretch in the ataxin-3 protein and subsequent neuronal death. The underlying intracellular signaling pathways are currently unknown. We applied the Reverse-phase Protein MicroArray (RPMA) technology to assess the levels of 50 signaling proteins (in phosphorylated and total forms) using three in vitro and in vivo models expressing expanded ataxin-3: (i) human embryonic kidney (HEK293T) cells stably transfected with human ataxin-3 constructs, (ii) mouse embryonic fibroblasts (MEF) from SCA3 transgenic mice, and (iii) whole brains from SCA3 transgenic mice. All three models demonstrated a high degree of similarity sharing a subset of phosphorylated proteins involved in the PI3K/AKT/GSK3/mTOR pathway. Expanded ataxin-3 strongly interfered (by stimulation or suppression) with normal ataxin-3 signaling consistent with the pathogenic role of the polyglutamine expansion. In comparison with normal ataxin-3, expanded ataxin-3 caused a pro-survival stimulation of the ERK pathway along with reduced pro-apoptotic and transcriptional responses.

## Introduction

Spinocerebellar ataxia type 3 (SCA3), also known as Machado-Joseph disease (MJD), is an autosomal dominantly inherited ataxia which is characterized by deficits in gait, movement, and coordination linked to a CAG repeat expansion in the *ATXN3* gene and a concordant polyglutamine expansion in the ataxin-3 protein. Therefore, SCA3 falls into the group of polyglutamine disorders which includes Huntington’s disease and within the broader category of neurodegenerative disorders to include Parkinson’s disease and Alzheimer’s disease [55].

Although the gene responsible for SCA3 was discovered more than 25 years ago [25], the cellular signaling pathways of neurodegeneration remain elusive. Protein activation mapping and identification of critical nodes in the signaling network represent central approaches required to elucidate the underlying nature of the disease and for the development of effective therapeutic interventions. Traditional low-throughput methods of protein analysis such as Western blotting and immunohistochemistry have served an important role in understanding SCA3, but their well-known limitations include difficulty in analyzing complex signaling networks at the level of protein expression and post-translational modification. On the other hand, DNA microarrays and RNA sequencing provide readouts restricted to the mRNA level with little follow-up on how these changes manifest at the protein level. While RNA encodes information about cellular status, proteins are the ultimate drivers of the cellular machinery serving in key mechanism of cell signaling through post-translational modifications. Reversible protein phosphorylation, especially on serine, threonine or tyrosine residues, is one of the most important and well-studied post-translational regulating protein function and signal transmission [5]. However, phosphorylated proteins are notoriously difficult to analyze on a mass scale by traditional methods such as Western blot especially when small amounts of samples need to be tested with high sensitivity.

To that end we utilized the Reverse-phase Protein MicroArray (RPMA) technology, a sensitive, quantitative, and high-throughput immunoassay to analyze protein and post-translational modification at the level of total protein abundance or specific phosphorylation. In the RPMA analysis a few microliters of cell lysates are printed onto nitrocellulose slides which are then probed with the protein-specific antibodies. The amount of bound antibody is quantitated using a highly sensitive colorimetric or fluorometric procedure [60].

Here, we present an RPMA-based analysis of three SCA3 models including human embryonic kidney (HEK293T) cells stably-transfected with plasmids encoding different ataxin-3 variants with normal (15 glutamines, HEK^15Q^) and expanded polyglutamine repeat (148 glutamines, HEK^148Q^), mouse embryonic fibroblasts (MEF) from ataxin-3 148Q (MEF^148Q^, [12]) and ataxin-3 knockout mice (MEF^KO^, [44]), and whole-brain samples from a SCA3 mouse model (CamKII/SCA3^77Q^, [11, 43, 48]). Our data provide assessment of the phosphoprotein signaling landscape contributing to the development of the disease phenotype in the context of different SCA3 models and suggest that the RPMA technology can be broadly applied to characterize neurodegenerative disorders thus assisting in biomarker identification and developing novel targets for therapy.

## Materials and methods

### Cell and Mouse models of SCA3

HEK293T cells were stably transfected with a plasmid expressing green fluorescent protein (GFP)-tagged ataxin-3 with 15 glutamines (HEK^15Q^), ataxin-3 with 148 glutamines (HEK^148Q^), or empty GFP plasmid (HEK^empty^) as control. Transfected cells were sorted by FACS and highly expressing cells were collected for analysis [48]. Mouse embryonic fibroblasts (MEF) were isolated as described in Hübener et al. [24] from the ataxin-3 148Q (MEF^148Q^), ataxin-3 knockout (MEF^KO^), and wild-type mice (MEF^wt^). The ataxin-3 knockout mouse line is described in Schmitt et al. [45] and the ataxin-3 148Q mouse line was generated as described in Boy et al. [12]. C57BL/6 wild-type mice were used to isolate control fibroblasts. The CamKII/SCA3^77Q^ mouse model is described in Schmidt et al. [44]. Mice were sacrificed at 12 months of age.

#### RPMA analysis

For Reverse-phase Protein MicroArray Analysis (RPMA, [60]) we used an established and pre-validated set of 50 different antibodies [38, 39] directed against the total and phosphorylated forms of signaling proteins which cover a wide array of signaling pathways relevant to the cell fate including apoptosis, survival, and autophagy (Additional file 1: Tables S1 and S2). All antibodies were validated for specificity prior to testing. RPMA analysis was performed according to Einspahr et al. [17]. Samples were printed onto the array slides at concentrations 100%, 50%, 25%, and 12.5% in duplicates and prepared for staining by treating with Reblot (Chemicon, CA). Slides were treated with blocking solution I-block (Applied Biosystems, MA) at 2 g/l and 0.5% Tween-20 in PBS. The total protein amounts loaded on the chip were estimated using SYPRO Ruby Protein Blot Stain (Invitrogen, CA) according to the manufacturer’s instructions and imaged on the NovaRay scanner (Alpha Innotech, CA). Blocked arrays were stained with antibodies on an automated slide stainer (Dako, CA) using the Catalyzed Signal Amplification System kit according to the manufacturer’s recommendation (Dako, CA). A signal was generated using streptavidin-conjugated IRDye 680 (LI-COR Biosciences, NE). Stained slides were scanned on the NovaRay scanner. The TIF images of the antibody and SYPRO-stained slides were analyzed using MicroVigene v2.9.9.9 software (VigeneTech, MA). Briefly, MicroVigene performed spot finding, local background subtraction (using local and slide average intensity), replicate averaging and total protein normalization, producing a single value for each sample at each dilution. All signal values produced for data analysis were at least two standard deviations above background. All four dilutions of each sample were analyzed for linear regression and only measurements which met linearity were kept for analysis. For each sample and antibody, SCA3 samples were normalized to the corresponding protein concentration in the wild-type sample at the same dilution and averaged across all available dilutions. These were then analyzed by the *t*-test to determine the p-value for each averaged protein level. All differences compared to wild-type found significant with 95% confidence are shown in Additional file 1: Table S1.

### Western blot analysis

Western blot analyses were essentially performed as described previously [48, 56] with 30 µg of protein loaded from each sample on Tris–glycine or Bis–Tris SDS polyacrylamide gels and separated electrophoretically. Afterwards, proteins were transferred to 0.2 µm nitrocellulose membranes (GE Healthcare, Dornstadt, Germany) using the respective transfer buffer. After blocking, the membranes were incubated with the same primary antibodies as used in the RPMA analysis (for details and dilutions see Additional file 1: Table S2) diluted in TBST at 4 °C overnight. Following the incubation with a fluorescence- or HRP-labeled secondary antibody, membranes were detected using an Odyssey Fc Imager (LI-COR Biosciences, Bad Homburg, Germany) and quantified using the Image Studio Software (LI-COR) or ImageJ. Statistical analyses were performed using GraphPad Prism 8.40 for Windows (GraphPad Software, San Diego, CA). Data is shown as arithmetic means ± SEM. Outliers were identified using Grubbs’ test with alpha = 0.05. Statistical significance of data sets was determined using Student’s *t*-test and p-values less than or equal to 0.05 were considered as statistically significant.

## Results

There is mounting evidence that inflammatory signaling cascades are involved in the development and pathogenesis of SCA3 [4, 15]. We thus generated a snapshot of the SCA3 degenerative landscape via a large screen of phosphorylated signaling proteins which act as principal regulators of signal transduction across multiple signaling networks. The list of tested proteins and their levels relative to corresponding controls are presented in Additional file 1: Table S1.

Our results show that the overexpression of both normal and expanded ataxin-3 has a profound effect on several signaling pathways. Of significant note is the phosphatidyl inositol 3-phosphate kinase (PI3K) cell survival pathway. Phosphorylation of its key members (Table 1) is robustly altered.

**Table 1:** Major pathways altered in SCA3 models

Pathway	Altered members
PI3K cell survival pathway	AKT, PTEN, p70S6K, GSK3, RPS6
Cell fate pathways	AKT, PTEN, JNK, ERK, GSK3, Src, p53, eNOS, p70S6K, RPS6, caspases 3, caspase 7, caspase 9
Apoptotic regulation	Bax, Bcl-xL, XIAP
Transcriptional regulation	STAT1, STAT3, IκBα
Pro-survival ERK-p70S6K pathway	ERK, p70S6K, RPS6
AKT/mTOR pathway targets	ERK, eNOS, GSK3, p53, p70S6K, PTEN, RPS6, Src

The table sums up the respective members of major pathways and pathway targets found to be altered in our RPMA analysis of SCA3 models

### Major signaling responses altered by expanded ataxin-3 in SCA3 models

**Fig. 1:**
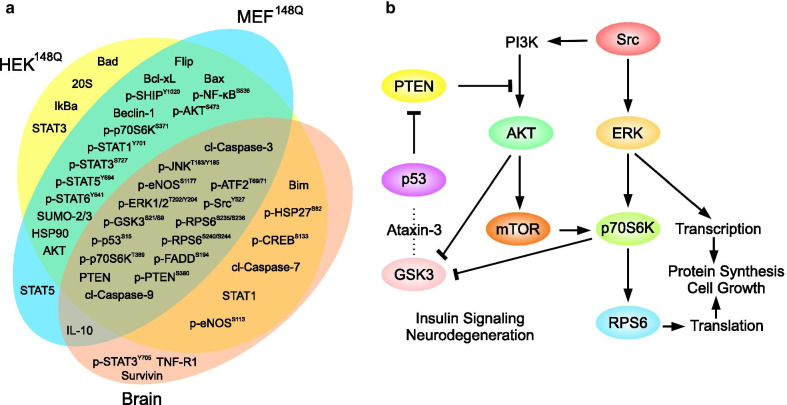
Altered signaling proteins in models of SCA3. **a** Signaling proteins responding to expanded ataxin-3 expression in the indicated SCA3 models (HEK^148Q^, MEF^148Q^ and brain of SCA3 mouse model) in comparison to the respective controls (HEK^wt^, MEF^wt^, and control brain). In all three different SCA3 disease models, we observed a significant overlap of dysregulated signaling proteins. For clarity, only the proteins displaying alterations of more that ± 20% relative to the corresponding control levels are shown. **b** A fragment of the signaling network illustrating connections between shared proteins in **a**

Although we applied a wide range of cellular and animal models, the sets of altered signaling proteins in response to expanded ataxin-3 were found to be rather similar (Fig. 1a). Pairwise comparison of the HEK^148Q^ and MEF^148Q^ cell responses in spite of the different species origin (human vs. mouse) revealed only four proteins as non-shared low responders (with levels changed by less than ± 0.1). As expected, brain of the SCA3 transgenic mouse model was more distant from the cultured cells: 16 responses were found unique for the MEF^148Q^ cells or the analyzed brains. All three models shared signaling nodes of major cell pathways important for cell fate (Table 1). A fragment of the signaling network illustrating these results is shown in Fig. 1b. Comparison of the HEK^15Q^ and HEK^148Q^ cells identified changes due to the polyglutamine expansion within ataxin-3 (Additional file 1: Table S1). Among the prominent differences (above the ± 20% threshold) between these cells the polyglutamine expansion caused reduction of the levels of apoptotic and transcriptional regulators (Table 1). The increased responses included the pro-survival ERK-p70S6K pathway (Table 1).

### Effect of wild-type ataxin-3 and the polyglutamine expansion

**Fig. 2:**
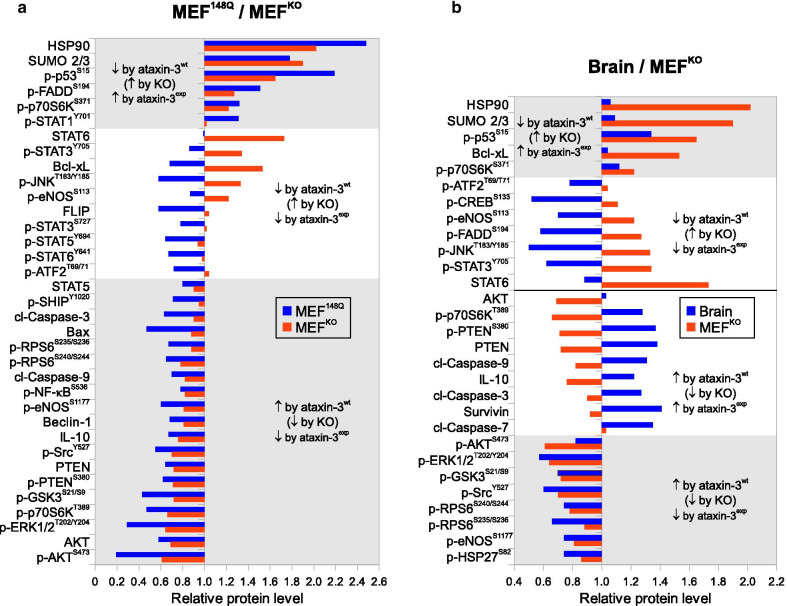
Side-by side comparison of signaling in reaction to the presence of wild-type and expanded ataxin-3 in MEF cells (**a**) and SCA3 mouse brain (**b**). Shown are the relative protein levels of signaling proteins altered upon the presence of wild-type ataxin-3 (comparison of MEF^KO^ with MEF^wt^) or expanded ataxin-3 (MEF^148Q^ vs. MEF^wt^) in MEF cells (**a**) as well as in mouse brain (SCA3 vs. wt mouse brain, **b**). Proteins are grouped according their stimulation (↑) / suppression (↓) by wild-type (ataxin-3^wt^) and expanded ataxin-3 (ataxin-3^exp^). For clarity, only the protein pairs displaying alterations of more that ± 20% in one or both proteins relative to the corresponding control levels are shown

To evaluate the contribution of normal (non-expanded) ataxin-3 in the cell signaling we analyzed cells lacking ataxin-3 (MEF^KO^) in comparison with wild-type cells (MEF^wt^). The proteins were grouped together based on their stimulation or suppression relative to the wild-type cells (Fig. 2). It is typically assumed that the proteins in the knock-out (KO) cells would display opposite effects (suppression *vs.* stimulation) compared to wild-type cells. We therefore compared the reaction towards the loss of ataxin-3 (MEF^KO^ vs. MEF^wt^) with the reaction induced by the polyglutamine expansion within ataxin-3 (MEF^148Q^ vs. MEF^wt^). Figure 2 shows that a number of responses induced by the presence of expanded ataxin-3 were suppressed by the presence of wild-type ataxin-3 (stimulated in MEF^KO^ cell). On the other hand, a large group of responses including the major cell fate proteins were stimulated by the presence of wild-type ataxin-3 (suppressed in MEF^KO^ cells) but suppressed in cells expressing expanded ataxin-3. No responses stimulated by wild-type ataxin-3 were found to be further amplified by the presence of expanded ataxin-3. As expected, the SCA3 whole brain samples demonstrated a certain degree of specificity compared to the MEF^148Q^ cells (Fig. 2). In spite of these specifics the results from both models demonstrated that the expanded ataxin-3 strongly interfered with the normal ataxin-3 signaling.

**Fig. 3:**
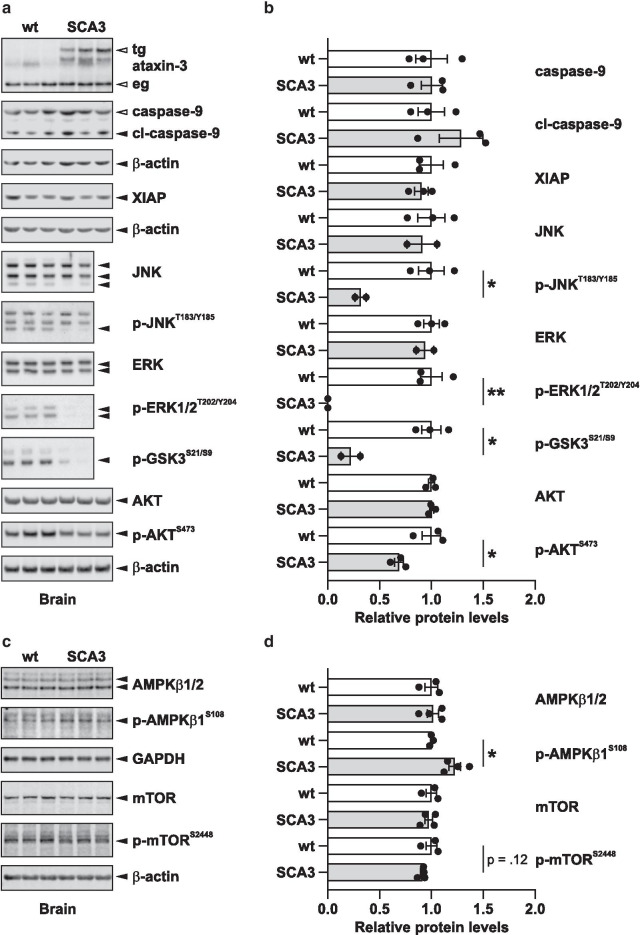
Confirmation of RPMA data by Western blot analysis. For validation of RPMA data, Western blot analyses of mouse brains of SCA3 and wild-type mice were performed for selected targets. The same antibodies as in the RPMA analysis were used. Blot images (**a**) and graphs (**b**) showing their quantification. Quantified bands are marked with a black arrow. β-actin was used as loading control. In the SCA3 mouse model, transgenic expanded ataxin-3 (tg) is detected in addition to the endogenous ataxin-3 (eg). In addition, total and phosphorylated levels of AMPKβ1 and mTOR were analyzed (**c** and **d**) to further dissect the impact of expanded ataxin-3 on the mTOR signaling pathway. The bars correspond to means (wt n = 3, SCA3 n = 2–4) ± SEM. Statistical significant differences (*t*-test) are marked (*, p < 0.05; **, p < 0.01)

To corroborate the results of the RPMA analysis, we validated a selection of marker proteins found dysregulated in the mouse brain samples by Western blot, demonstrating a strong consistency with our high-throughput approach (Fig. 3a, b). This includes the downregulation of the phosphorylated forms of AKT, ERK, GSK3 and JNK. To further dissect the apparently downregulated AKT/mTOR pathway, we investigated additional proteins namely total and phosphorylated levels of mTOR itself and its upstream inhibitor AMPKβ1. Western blot analysis showed a trend towards a reduction of the AKT and p70S6K-mediated activating phosphorylation of mTOR at Ser2448, and significantly increased levels of pSer108-AMPKb1 (Fig. 3c, d), both confirming rather inhibitory effects on the mTOR pathway by the expression of expanded ataxin-3 [21,23].

## Discussion

### Polyglutamine expanded ataxin-3 decreases pro-survival signaling via the AKT pathway

The serine/threonine-protein kinase AKT is a key protein kinase involved in survival and growth with physiological links to neurological disorders. The levels of Serine 473-phosphorylated AKT (p-AKT^S473^), representing an active form of this protein, were decreased in all three models indicating a drop in pro-survival signaling due to the 148Q expansion. AKT signaling cascades include a number of proteins previously implicated in SCA3 such as GSK3, FOXO and mTOR [4, 18, 27, 42]. An increased susceptibility towards oxidative stress (via FOXO4 and SOD2) was suggested to be a potential contributor to neuronal cell death in SCA3 and AKT, ERK, and JNK are all among the known kinases which target FOXOs and contribute to their activation and localization [4, 27].

Upstream of AKT lies the PI3K pathway. This pathway is balanced and activated by the tumor suppressor phosphatase and tensin homolog PTEN. In our analyses, we observed an increase of both the total and phosphorylated levels of PTEN in SCA3 mouse brain samples and a decrease in HEK293T and MEF cells expressing expanded ataxin-3. Interestingly, ataxin-3 is suggested to be responsible for repressing PTEN transcription in cancer [42] which further emphasizes the importance of the AKT signaling pathway in disease progression.

### Altered GSK3 signaling in SCA3 models

Importantly, we also saw a reduction of phosphorylated glycogen synthase kinase 3 (GSK3) shared among all SCA3 models investigated. GSK3α and GSK3β are constitutively active, proline-directed serine/threonine kinases involved in a variety of cellular processes including glycogen metabolism [58], gene transcription [50], apoptosis [51] and microtubule stability [3, 13]. GSK3 is known to play a role in several neurological disorders [30]. With respect to SCA3 it is known to phosphorylate ataxin-3 and thus regulate its aggregation propensity [19, 37]. Furthermore, the phosphorylation of ataxin-3 by GSK3 contributes to its nuclear translocation [37], a process increasing the toxicity of expanded ataxin-3 and required for the manifestation of the disease [9, 48]. The reduction of GSK3 may therefore reflect a protective mechanism against this increase of toxicity.

GSK3 was also the first reported substrate of AKT and the two are functionally similar. GSK3 is a strong promoter of Toll-like receptors-induced production of proinflammatory cytokines such as IL-6 which was found to be altered in SCA3 patient samples [18]. AKT is known to exert an inhibitory role on GSK3 via phosphorylation of Ser21/Ser9 (p-GSK3^S21/S9^) [22]. Consistent with the observed decrease in p-AKT^S473^, we detected a reduced inhibitory phosphorylation of GSK3 in HEK293T and MEF cells expressing expanded ataxin-3.

Furthermore, GSK3 is required for activation of STAT proteins in astrocytes, microglia and macrophages [8]. Accordingly, our SCA3 cell models showed changes in seven proteins compared to two STAT proteins in the brain samples, while MEF^KO^ cells showed profound alterations of STAT levels in response to the absence of ataxin-3 (Additional file 1: Table S1 and Fig. 2). These observations reinforce the link between ataxin-3 and GSK3 and should prompt the field to consider the role of insulin and glucose in brain homeostasis and neuronal degeneration as a metabolic syndrome. It is noteworthy, that GSK3 inhibitors recently gained attention in terms of their potential for diabetes, cancer, and neurodegeneration [33] highlighting a therapeutic potential for SCA3.

### SCA3 models exhibit alterations of AKT-associated factors

Tumor suppressor protein p53 is considered the “guardian of the genome” [28] because of its important role in determining cell fate. A tight regulation of p53 is critical in maintaining homeostasis and keeping a balance between its cancer-suppressive and age-promoting functions [10]. The interaction between p53 and GSK3 is hypothesized to be involved in aggregate clearance and neurodegeneration: the Alzheimer’s disease hypothesis suggests that under normal conditions, GSK3 is involved in keeping p53 levels low while under conditions of cellular stress (e.g. aggregation) p53 is stabilized, proteasomal function declines, and levels of p53 increase [40, 51]. We observed that the levels of activated p-p53^S15^ increased in MEF^148Q^ cells and in SCA3 mouse brains in contrast to its suppression by wild-type ataxin-3 (increase in MEF^KO^ cells). Recently, p53 was identified as a substrate of wild-type ataxin-3 and the polyglutamine expansion was associated to increased p53-dependent neuronal death [31]. One can hypothesize that SCA3 and other neurodegenerative disorders involve a positive feedback between proteasomal degradation, aggregation, GSK3 activation, and increased p53 levels. Besides p53, the strongest increase in MEF cells both as reaction to the expression of expanded ataxin-3 and to the loss of ataxin-3 were observed for HSP90 and SUMO 2/3. Both proteins were implicated before in SCA3 [2, 47] and our results further stress their relevance. Ataxin-3 is SUMOylated and the SUMOylation of ataxin-3 regulates its function [2]. Moreover, the inhibition of HSP90 turned out as promising therapeutic strategy in a mouse model for SCA3 [47].

The extracellular signal regulated kinase ERK is a member of the MAPK pathway. It is a key protein lying at the intersection of proliferation, differentiation, and survival [29]. It was previously shown that ERK is responsible for the calcium-dependent phosphorylation of ataxin-1 [26] and the downregulation of ERK reduces levels of ataxin-1 and suppresses neurodegeneration in *Drosophila* and mice [20]. ERK is further able to act on downstream pathways of AKT and mTOR via control of the p70S6 kinase and the S6 ribosomal subunit [34]. The phosphorylated ERK (p-ERK1/2^T202/Y204^) was reduced in all models tested including the knockout MEF^KO^ cells. The latter suggested that the stimulating effect of wild-type ataxin-3 in regulating p-ERK1/2^T202/Y204^ levels was abrogated by the polyglutamine expansion. We also found that similar to ERK, another MAPK, stress-activated p-JNK^T183/Y185^ displayed a strong inhibition by the expanded ataxin-3.

An additional target of AKT is the endothelial nitric oxide synthase (eNOS) which catalyzes the production of nitric oxide. We observed a decrease in eNOS activation by ataxin-3 148Q in all models similar to ERK. The phosphorylation of eNOS at Ser1177 (p-eNOS^S1177^) by AKT activates this enzyme. In terms of neurodegeneration, alterations in eNOS have been linked to blood–brain barrier integrity [6] which directly points to the importance of peripheral inflammation in propagation of CNS dysfunction.

### Indication of impaired mTOR activation in SCA3 models

P70S6 kinase is a mitogen-activated kinase affected by ERK signaling downstream of mTOR and AKT. It phosphorylates the S6 protein of the 40S ribosomal subunit (RPS6) and thus controls mRNA translation. The phosphorylation of RPS6 is commonly used as a readout of mTOR activation [36] and other work suggests that the state of Ser240/Ser244 phosphorylation of RPS6 (p-RPS6^S240/S244^) can be used to estimate the neuronal activity state of striatal cholinergic interneurons [7]. We observed decreased levels of p-RPS6^S240/S244^ in all models and MEF^KO^ cells indicating a negative impact of the presence of expanded ataxin-3 on mTOR activation. Moreover, an interaction between ataxin-3 and the kinase (ribosomal protein S6 kinase alpha-1) phosphorylating RPS6 has also been suggested [1, 52].

Along with p70S6K, another important neuron-related kinase connected with mTOR is Src. Our data indicate an increased activity of Src in our models: The Tyr527 phosphorylation site of Src (p-Src^Y527^) which renders the enzyme less active was decreased in HEK^148Q^, MEF^148Q^ and mouse brain samples. Although first identified as a proto-oncogene, Src was found to be expressed in differentiated, post-mitotic neurons and is important for neuronal differentiation and neurite outgrowth with the capability to control ion channel activity and synaptic transmission [43]. Src is involved in the intracellular release of calcium stores from the endoplasmic reticulum [53]. Concordant with our data, a reduced phophorylation of Src has also been observed in a different mouse model of SCA3 [59]. Moreover, Src has been linked to Huntington’s disease where expression of polyglutamine-expanded huntingtin activates Src and ultimately promotes neuronal death induced by glutamate. In this line, Src was found to be altered in ataxin-2 patient fibroblasts and ataxin-2 knockout lines [16].

Further supporting our RPMA analysis, we observed using Western blots a trend towards reduced phosphorylation of mTOR at Ser2448, an activating modification mediated by AKT and p70S6K [23], and significantly increased levels of Ser108-phosphorylated AMPKβ1, which acts as an upstream inhibitor of mTOR [21, 54]. These observations support the indication of rather impaired mTOR activation. In an earlier study, the inhibition of mTOR led to an induction of autophagy thereby ameliorating the toxicity of expanded ataxin-3 in SCA3 mice [35]. Consistently, increasing phosphorylated AMPK by the administration of cordycepin in SCA3 cells and mice was shown to have neuroprotective effects via activation of autophagy and lowering mutant protein synthesis [32]. Thus, these findings suggest that the detected lowering of mTOR activation in our models may represent a rescue mechanism.

### Pro-survival, anti-apoptotic contribution of polyglutamine expansion

Apoptosis and necrosis are believed to be the two major death pathways for neurons [14]. Regarding apoptosis, it was suggested that initiator or executor caspases are activated in neurodegenerative diseases [57]. In Huntington’s disease, neurons positive for caspase-3 die quickly but for those with aggregates trigger cellular quiescence, deactivate apoptosis but activate delayed necrosis, which supports the argument that high-ordered aggregates or inclusions might be protective [41]. In our analysis, the levels of cleaved caspases including caspase-3, -7, and -9 were increased in SCA3 mouse brains while either decreased or not altered in MEF and HEK293T cell models. Seemingly contradictory behavior was displayed by the pro-apoptotic FADD (activated in MEF^148Q^ cells, but suppressed in our other models). Bcl-2 family members (Bcl-xL, Bax, Bad, Bim) as well as FLIP, SHIP and XIAP were uniformly either reduced or not altered. Recent data showed that the ratio of Bcl2/Bax was decreased in SCA3 patients compared to controls, suggesting that the ratios and balances of these proteins are perhaps more important than their absolute values [61]. Consistent with this, comparison of normal vs. expanded ataxin-3 showed that the presence of the polyglutamine expansion within ataxin-3 provided a pro-survival and anti-apoptotic contribution. Since the cell behavior is dictated by a superposition of multiple signals, the overall balance in favor of survival or death outcomes cannot be predicted with certainty based on our data only. Nevertheless, it seems reasonable to conclude that, depending on the particular cellular context, the expanded ataxin-3 can provide a widespread anti-apoptotic impact on cell signaling.

## Conclusion

In our work, we used RPMA analysis to characterize the altered signaling networks observed upon ataxin-3 expansion in both in vitro and in vivo models. It is important to consider that these models have large differences such as innate expression of ataxin-3, overexpression of ataxin-3, have different levels of sensitivity to expanded protein and are derived from different tissues. We employed a set of antibodies which cover various pathways across the cellular landscape including inflammation, survival, energy metabolism, growth, transcription, translation, apoptosis, mitochondrial integrity and autophagy. We suggest that elucidating these signaling cascades is integral in understanding the function of ataxin-3 in health and disease. We demonstrate that across our models, AKT/mTOR pathway targets (Table 1) are significantly altered. Our results revealed alterations shared between tested models as well as the unique differences providing new perspectives on altered signaling in SCA3. In future studies, the role of the altered pathways needs to be correlated to the disease progression and outcome in SCA3 patients and animal models [46, 49]. This analysis can shed light on future targets for research and pharmaceutical development by painting a clearer picture of the neurodegenerative landscape.

## Supplementary information

**Additional file 1: Table S1.** RPMA analysis of relative protein levels.** Table S2.** Used antibodies and dilutions.

## Data Availability

All data generated or analyzed during this study are included in this published article and its supplementary information.

## Abbreviations

SCA3: Spinocerebellar ataxia type 3
MJD: Machado-Joseph disease
AKT: AKT8 virus oncogene cellular homolog
ATF: Activating transcription factor 2
Bad: Bcl-2-associated agonist of cell death
Bax: Bcl-2-associated X protein
Bcl-xL: B-cell lymphoma-extra large
Bim: Bcl-2-interacting mediator
cl: Prefix cl- denotes a cleaved form
CREB: CAMP response element-binding protein
eNOS: Endothelial nitric oxide synthase
ERK: Extracellular signal-regulated kinase
FADD: Fas-associated protein with death domain
FLIP: FLICE (Caspase-8) inhibitory protein
FOXO: Forkhead box protein class O4
GAPDH: Glyceraldehyde-3-phosphate dehydrogenase
GSK: Glycogen synthase kinase
HSP27: Heat shock protein 27
HSP90: Heat shock protein 90
IκBα: Inhibitor of NF-κB alpha subunit
IL-10: Interleukin 10
JNK: Jun N-terminal kinase
mTOR: Mammalian target of rapamycin
NF-κB: Nuclear factor κB
p-: Prefix p- denotes a phosphorylated protein
p53: Tumor suppressor protein that protects from DNA damage
p70S6K: Phosphoprotein 70 ribosomal protein S6 kinase
PARP: Poly(ADP-ribose) polymerase
PTEN: Phosphatase and tensin homolog deleted on chromosome 10
RPMA: Reverse-phase Protein MicroArray
RPS6: Small-subunit ribosomal protein S6
SHIP: Src homology 2 (SH2) domain-containing inositol phosphatase
Src: Rous sarcoma oncogene cellular homolog
STAT: Signal transducer and activator of transcription
SUMO: Small ubiquitin-like modifier
TNF-R1: Tumor necrosis factor receptor 1
wt: Wild-type
XIAP: X-linked inhibitor of apoptosis
