# Supplementary material for: Neurodegenerative phosphoprotein signaling landscape in models of SCA3
Source: Mol Brain. 2021 Mar 19;14:57. doi: 10.1186/s13041-020-00723-0 (PMC7980345; doi:10.1186/s13041-020-00723-0)
Supplement: Supplementary file 1 — Additional file 1: Table S1. RPMA analysis of relative protein levels. Table S2. Used antibodies and dilutions. [file 13041_2020_723_MOESM1_ESM.docx]

**Additional file 1: Table S1. RPMA analysis of relative protein levels**

Shown are comparisons between HEK293T cells expressing normal ataxin-3 with 15Q (HEK^15Q^), expanded ataxin-3 with 148Q (HEK^148Q^), control cells transfected with an empty vector (HEK^empty^), mouse embryonic fibroblasts (MEF) isolated from ataxin-3 148Q mice (MEF^148Q^), ataxin-3 knockout mice (MEF^KO^) and wild-type mice (MEF^wt^) as well as brain samples from the CamKII/SCA3^77Q^ mouse model (Mouse SCA3) and wild-type mice (wt). All samples were run in duplicates. Each duplicate was run in four dilutions. All values are means with p<0.05 normalized to total protein concentration. ND, not determined.

| **Proteins** | **HEK^15Q^ vs. HEK^empty^** | **HEK^148Q^ vs. HEK^empty^** | **HEK^148Q^ vs. HEK^15Q^** | **MEF^148Q^ vs. MEF^wt^** | **MEF^KO^ vs. MEF^wt^** | **Mouse SCA3 vs. wt** |
| --- | --- | --- | --- | --- | --- | --- |
| AKT | 0.73 | 0.68 | 0.94 | 0.58 | 0.69 | 1.03 |
| p-AKT^S473^ | 0.63 | 0.59 | 0.94 | 0.19 | 0.61 | 0.82 |
| p-ATF2^T69/T71^ | 1.07 | 1.23 | 1.14 | 0.72 | 1.04 | 0.78 |
| Bad | 0.77 | 0.74 | 0.97 | 0.91 | 1.05 | 0.98 |
| Bax | 0.83 | 0.61 | 0.73 | 0.47 | 0.88 | 1.03 |
| Bcl-xL | 0.78 | 0.53 | 0.69 | 0.68 | 1.53 | 1.04 |
| Beclin-1 | 0.78 | 0.70 | 0.89 | 0.68 | 0.81 | 0.97 |
| Bim | 0.78 | 0.67 | 0.86 | ND | ND | 0.79 |
| cl-Caspase-3 | 0.66 | 0.61 | 0.93 | 0.63 | 0.90 | 1.27 |
| cl-Caspase-7 | 0.69 | 0.71 | 1.03 | 0.81 | 1.03 | 1.35 |
| cl-Caspase-9 | 0.67 | 0.66 | 0.99 | 0.70 | 0.82 | 1.31 |
| p-CREB^S133^ | 0.80 | 0.73 | 0.91 | 0.89 | 1.11 | 0.52 |
| p-eNOS^S113^ | 0.74 | 0.60 | 0.81 | 0.87 | 1.22 | 0.70 |
| p-eNOS^S1177^ | 0.77 | 0.69 | 0.90 | 0.60 | 0.81 | 0.74 |
| p-ERK1/2^T202/Y204^ | 0.47 | 0.67 | 1.43 | 0.29 | 0.64 | 0.57 |
| p-FADD^S194^ | 0.67 | 0.62 | 0.94 | 1.51 | 1.27 | 0.58 |
| FLIP | 0.82 | 0.72 | 0.88 | 0.58 | 1.04 | 1.05 |
| p-GSK3^S21/S9^ | 0.58 | 0.61 | 1.04 | 0.43 | 0.72 | 0.70 |
| p-HSP27^S82^ | 1.22 | 1.26 | 0.94 | ND | 0.86 | 0.74 |
| HSP90 | 0.85 | 0.74 | 0.87 | 2.48 | 2.02 | 1.06 |
| IκBα | 0.71 | 0.50 | 0.71 | 1.11 | 1.07 | 1.07 |
| IL-10 | 1.13 | 0.97 | 0.85 | 0.67 | 0.76 | 1.22 |
| p-JNK^T183/Y185^ | 0.73 | 0.77 | 1.05 | 0.58 | 1.33 | 0.50 |
| p-NF-κB^S536^ | 0.64 | 0.67 | 1.05 | 0.78 | 0.82 | 1.08 |
| p-p53^S15^ | 0.66 | 0.70 | 1.05 | 2.19 | 1.65 | 1.34 |
| p-p70S6K^S371^ | 0.79 | 0.78 | 0.99 | 1.32 | 1.22 | 1.12 |
| p-p70S6K^T389^ | 0.10 | 0.22 | 2.22 | 0.47 | 0.66 | 1.28 |
| cl-PARP | 0.72 | 0.84 | 1.17 | ND | ND | 0.95 |
| 20S | 0.78 | 0.34 | 0.41 | 0.92 | 0.94 | 1.03 |
| PTEN | 0.71 | 0.65 | 0.91 | 0.64 | 0.72 | 1.38 |
| p-PTEN^S380^ | 0.72 | 0.66 | 0.92 | 0.62 | 0.71 | 1.37 |
| p-RPS6^S235/236^ | 0.25 | 0.27 | 1.07 | 0.67 | 0.88 | 0.66 |
| p-RPS6^S240/S244^ | 0.31 | 0.43 | 1.42 | 0.65 | 0.78 | 0.74 |
| p-SHIP^Y1020^ | 0.81 | 0.65 | 0.80 | 0.71 | 0.95 | 1.04 |
| p-Src^Y416^ | 1.10 | 1.28 | 1.17 | ND | ND | 0.94 |
| p-Src^Y527^ | 0.69 | 0.68 | 0.99 | 0.55 | 0.70 | 0.60 |
| STAT1 | 0.72 | 0.63 | 0.87 | 1.09 | 1.06 | 1.21 |
| p-STAT1^Y701^ | 0.80 | 0.57 | 0.72 | 1.31 | 1.02 | 1.05 |
| STAT3 | 0.81 | 0.54 | 0.67 | 0.87 | 0.99 | 1.02 |
| p-STAT3^Y705^ | 1.09 | 1.00 | 0.92 | 0.86 | 1.34 | 0.62 |
| p-STAT3^S727^ | 0.78 | 0.53 | 0.69 | 0.78 | 1.02 | 1.01 |
| STAT5 | 0.90 | 0.82 | 0.92 | 0.80 | 0.90 | 1.14 |
| p-STAT5^Y694^ | 0.68 | 0.63 | 0.93 | 0.64 | 0.94 | 0.98 |
| STAT6 | 1.29 | 0.93 | 0.73 | 0.99 | 1.73 | 0.88 |
| p-STAT6^Y641^ | 0.89 | 0.72 | 0.81 | 0.67 | 0.98 | 0.88 |
| SUMO-1 | 0.76 | 0.71 | 0.93 | ND | ND | 0.97 |
| SUMO-2/3 | 0.73 | 0.70 | 0.95 | 1.78 | 1.90 | 1.09 |
| Survivin | ND | ND | ND | 1.05 | 0.92 | 1.41 |
| TNF-R1 | ND | ND | ND | ND | ND | 0.16 |
| XIAP | 0.80 | 0.58 | 0.73 | ND | ND | 1.02 |

**Additional file 2: Table S2. Used antibodies and dilutions**

Listed are the primary antibodies used in the Reverse-Phase Microarray Analysis (RPMA) and the confirmatory Western blots. The antigens as well as the designations used in this manuscript, the vendor (Cell Signaling Technology (CellSignal) or abcam), the product ID and the dilution(s) used in the RPMA or western blot assay are listed.

| **Antigen** | **Designation** | **Vendor** | **Product #** | **Dilution** |
| --- | --- | --- | --- | --- |
| AKT | AKT | CellSignal | 9272 | 1:100/1:1,000 |
| phospho-AKT (Ser473) | p-AKT^S473^ | CellSignal | 9271 | 1:100/1:1,000 |
| AMPKβ1/2 | AMPKβ1/2 | CellSignal | 4150 | 1:500 |
| phospho-AMPKβ1 (Ser108) | p-AMPKβ1^S108^ | CellSignal | 4181 | 1:500 |
| phospho-ATF-2 (Thr69/Thr71) | p-ATF2^T69/T71^ | CellSignal | 9225 | 1:500 |
| Bad | Bad | CellSignal | 9292 | 1:100/1:1,000 |
| Bax | Bax | CellSignal | 2772 | 1:1,000 |
| Bcl-xL | Bcl-xL | CellSignal | 2762 | 1:500 |
| Beclin-1 | Beclin-1 | CellSignal | 3738 | 1:250 |
| Bim | Bim | CellSignal | 2933 | 1:1,000 |
| cleaved Caspase-3 (Asp175) | cl-Caspase-3 | CellSignal | 9661 | 1:50 |
| cleaved Caspase-7 (Asp198) | cl-Caspase-7 | CellSignal | 9491 | 1:50 |
| cleaved Caspase-9 (Asp330) | cl-Caspase-9 | CellSignal | 9501 | 1:100/1:1,000 |
| phospho-CREB (Ser133) | p-CREB^S133^ | CellSignal | 9191 | 1:100 |
| phospho-eNOS (Ser113) | p-eNOS^S113^ | CellSignal | 9575 | 1:50 |
| phospho-eNOS (Ser1177) | p-eNOS^S1177^ | CellSignal | 9571 | 1:50 |
| phospho-p44/42 MAPK (ERK1/2) (Thr202/Tyr204) | p-ERK1/2^T202/Y204^ | CellSignal | 9101 | 1:500/1:5,000 |
| phospho-FADD (Ser194) | p-FADD^S194^ | CellSignal | 2781 | 1:200 |
| FLIP | FLIP | CellSignal | 3210 | 1:100 |
| GAPDH | GAPDH | abcam | 125247 | 1:2000 |
| phospho-GSK-3α/β (Ser21/Ser9) | p-GSK3^S21/S9^ | CellSignal | 9331 | 1:250 |
| phospho-HSP27 (Ser82) | p-HSP27^S82^ | CellSignal | 2406 | 1:100 |
| HSP90 | HSP90 | CellSignal | 4875 | 1:500 |
| IκBα | IκBα | CellSignal | 9242 | 1:500 |
| Interleukin 10 | IL-10 | abcam | 52909 | 1:2,000 |
| phospho-SAPK/JNK (Thr183/Tyr185) | p-JNK^T183/Y185^ | CellSignal | 9251 | 1:500 |
| mTOR | mTOR | CellSignal | 2972 | 1:500 |
| phospho-mTOR (Ser2448) | mTOR^S2448^ | CellSignal | 2971 | 1:500 |
| phospho-NF-κB p65 (Ser536) | p-NF-κB^S536^ | CellSignal | 3033 | 1:50 |
| phospho-p53 (Ser15) | p-p53^S15^ | CellSignal | 9284 | 1:100 |
| phospho-p70 S6 Kinase (Ser371) | p-p70S6K^S371^ | CellSignal | 9208 | 1:50 |
| phospho-p70 S6 Kinase (Thr389) | p-p70S6K^T389^ | CellSignal | 9205 | 1:50 |
| cleaved PARP (Asp214) | cl-PARP | CellSignal | 9541 | 1:200 |
| Proteasome 20S C2/HC2 (α6) subunit | 20S | abcam | 3325 | 1:2,000 |
| PTEN | PTEN | CellSignal | 9552 | 1:500 |
| phospho-PTEN (Ser380) | p-PTEN^S380^ | CellSignal | 9551 | 1:200 |
| phospho-S6 Ribosomal Protein (Ser235/236) | p-RPS6^S235/236^ | CellSignal | 4856 | 1:200 |
| phospho-S6 Ribosomal Protein (Ser240/Ser244) | p-RPS6^S240/S244^ | CellSignal | 2215 | 1:1,000 |
| phospho-SHIP1 (Tyr1020) | p-SHIP^Y1020^ | CellSignal | 3941 | 1:1,000 |
| phospho-Src (Tyr416) | p-Src^Y416^ | CellSignal | 2101 | 1:250 |
| phospho-Src (Tyr527) | p-Src^Y527^ | CellSignal | 2105 | 1:250 |
| STAT1 | STAT1 | CellSignal | 9172 | 1:100 |
| phospho-STAT1 (Tyr701) | p-STAT1^Y701^ | CellSignal | 9171 | 1:1,000 |
| STAT3 | STAT3 | CellSignal | 9132 | 1:1,000 |
| phospho-STAT3 (Tyr705) | p-STAT3^Y705^ | CellSignal | 9131 | 1:100 |
| phospho-STAT3 (Ser727) | p-STAT3^S727^ | CellSignal | 9134 | 1:100 |
| STAT5 | STAT5 | CellSignal | 9358 | 1:200 |
| phospho-Stat5 (Tyr694) | p-STAT5^Y694^ | CellSignal | 9354 | 1:50 |
| STAT6 | STAT6 | CellSignal | 9362 | 1:200 |
| phospho-STAT6 (Tyr641) | p-STAT6^Y641^ | CellSignal | 9361 | 1:100 |
| SUMO-1 | SUMO-1 | CellSignal | 4930 | 1:200 |
| SUMO-2/3 | SUMO-2/3 | CellSignal | 4971 | 1:2,000 |
| Survivin | Survivin | CellSignal | 2808 | 1:500 |
| TNF-R1 | TNF-R1 | CellSignal | 3736 | 1:500 |
| XIAP | XIAP | CellSignal | 2042 | 1:100 |
